# Case Report: Severe COVID-19 with Late-Onset Sepsis-like Illness in a Neonate

**DOI:** 10.4269/ajtmh.21-0743

**Published:** 2022-02-15

**Authors:** Suryadi Nicolaas Napoleon Tatura

**Affiliations:** Division of Pediatric Infection and Tropical Medicine, Department of Pediatrics, Faculty of Medicine, Sam Ratulangi University, Manado, Indonesia; Department of Pediatrics, Prof. R. D. Kandou General Hospital, Manado, North Sulawesi, Indonesia; Department of Pediatrics, Robert Wolter Mongisidi Army Hospital, Manado, North Sulawesi, Indonesia

## Abstract

A case of severe COVID-19 with late-onset sepsis-like illness is presented in a neonate. A male infant was born to a mother with mild COVID-19 symptoms and positive IgG anti-severe acute respiratory syndrome coronavirus 2 (SARS-CoV-2) through spontaneous vaginal delivery. He and his mother were then confirmed to have SARS-CoV-2 infection. His condition was stable and discharge from the hospital was planned. However, on day 6 of care, his condition deteriorated, and after treatment with the COVID-19 protocol and antibiotic administration (because neonatal sepsis had not been ruled out), his condition gradually improved and he was discharged in good clinical condition without any sequelae. The pitfalls of this case are the presence of late-onset severe COVID-19, and the difficulty in monitoring patients in isolation rooms, which makes it challenging to differentiate and manage therapy between severe COVID-19 and neonatal sepsis. The similarities between the presentation of sepsis and severe COVID-19 require a thorough anamnesis, careful observation, and a thorough workup for alternative causes of sepsis to be able to make wise antibiotics treatment decisions, to prevent mismanagement, and to reduce morbidity and mortality, especially in developing countries such as Indonesia.

## INTRODUCTION

From the beginning of the pandemic until August 2021, Indonesian data show that 6 of 1,000 Indonesian children age 0 to 18 years are positive for COVID-19 using a reverse transcription–polymerase chain reaction (RT-PCR) test, and neonates are a group that is prone to serious illness and death. ^1^^,^ ^2^ In addition, sepsis is still a health problem that has high morbidity and mortality in Asia, including Indonesia. ^3^^,^ ^4^

COVID-19 in pediatric patients is mostly mild or asymptomatic. ^5^ However, a previous study of neonates reported that in 25 cases of COVID-19, 80% of patients were symptomatic, and those who were severely ill included a greater proportion of neonates than children more than 1 month old (12% versus 2%). ^6^ Severe acute respiratory syndrome coronavirus 2 (SARS‐CoV‐2) spreads rapidly in children, suggesting that it has a strong transmission capacity in neonates. ^7^ Neonates are thought to be more susceptible to SARS-CoV-2 because their immune system is not well developed. ^8^ Some studies reported that the most common symptoms of neonates with confirmed COVID-19 were respiratory distress, fever, and feeding intolerance, such as in neonatal pneumonia. ^7^ Similar to other viruses, such as enteroviral and herpes simplex virus infections, the infected neonates with this condition will have clinical presentations similar to neonatal sepsis caused by bacteria, and it can be very challenging to distinguish among them. ^9^^–^ ^11^ Therefore, it is important to do a thorough investigations for bacterial causes in every confirmed COVID-19 case. To the best of our knowledge, this is the first reported case of COVID-19 that presented with late-onset neonatal sepsis.

## CASE PRESENTATION

**Figure 1.**
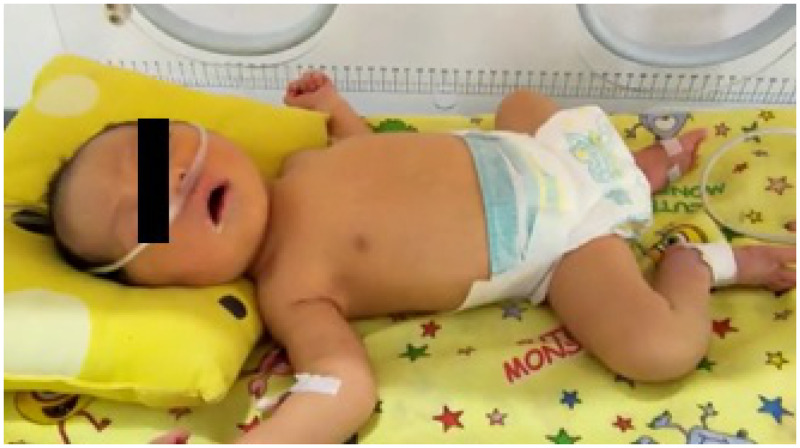
Photo of the patient on day 6 of hospitalization (icteric, Kramer III). This figure appears in color at www.ajtmh.org.

**Figure 2.**
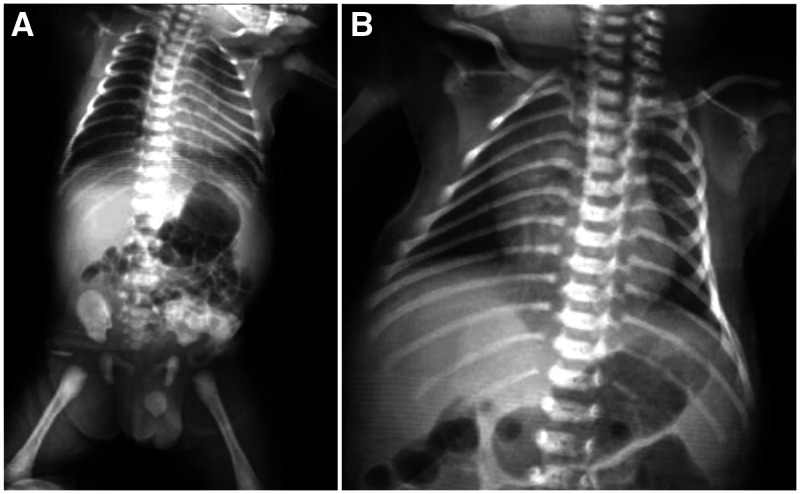
(**A**) Chest X-ray on day 1 of hospitalization is normal. (**B**) On day 6 of hospitalization, the chest X-ray indicates right pneumonia.

A male infant was born on November 29, 2020 from a mother with suspected COVID-19 infection based on positive serological confirmation with positive IgG anti-SARS-CoV-2 of the mother before delivery at Wolter Mongisidi Army Hospital, Manado. The mother was confirmed to have SARS-COV-2 by RT-PCR on the day after delivery. The infant was born via spontaneous vaginal delivery, at a gestational age of 37 to 38 weeks. His birth weight was 3,200 g and his length was 48 cm. The newborn’s Apgar score at minute 1 was 7 points and at minute 5 was 9 points. After birth, the newborn cried immediately, appeared active, and had no shortness of breath. The temperature of the newborn was 36.9°C, with a respiratory rate of 40 cycles/min, a pulse rate of 146 beats/min, and a saturated oxygen level of 99%. Other physical examination findings were normal. The blood result on the first day of life was hemoglobin, 16.8 mg/dL; hematocrit, 49.2%; leukocyte count, 9,800 leukocytes/µL; thrombocyte count, 285,000 thrombocytes/µL; neutrophile-to-lymphocyte ratio, 3.6; total lymphocyte count, 2,116 lymphocytes, and C-reactive protein, < 6 mg/dL. Chest radiography showed normal results (Figure 1). The next day (< 24 hours), the patient was tested for SARS-CoV-2, and the result of RT-PCR examination was positive on day 3. He was diagnosed as a term infant with an appropriate gestational age, with confirmed COVID-19, and was asymptomatic. This patient’s status was categorized as congenital COVID-19 based on positive serological confirmation with positive IgG anti-SARS-CoV-2 of the mother before delivery and a positive RT-PCR SARS-CoV-2 result in the infant. The mother and newborn shared the same room. The mother wore a mask and performed hand hygiene when performing hands-on care. When not taking care of her baby directly, she maintained a physical distance from her infant of more than 2 m. The newborn was fed milk formula because the mother’s milk was still lacking during the first 3 days after delivery. The baby’s weight decreased from 3,200 g at birth to 3,000 g on day 3. In general, infants born spontaneously who are in good condition are treated for 1 day only. However, because the protocol required screening, mother and baby were discharged on day 3 of care and were cautioned to self-isolate at home because the infant was in good clinical condition with a normal chest X-ray. Unfortunately, the infant was not able to go home because there was no place qualified for self-isolation in the home. Therefore, he was placed in an isolation room. From days 1 through 4, the patient was stable, in good condition, and was observed in the isolation room. On day 5, he became jaundiced, but remained in a stable state. His mother has no history of hepatitis. On day 6, the patient deteriorated and developed dyspnea, fever, and looked icteric with Kramer III during examination (Figure 1). In addition, physical examination revealed subcostal and intercostal retractions. The blood chemistry test results were as follows: total bilirubin, 13.60 mg/dL; direct bilirubin, 1.00 mg/dL; and indirect bilirubin, 12.60 mg/dL (Table 1). Chest radiography revealed right pneumonia (Figure 2). He was diagnosed as a term infant with an appropriate for gestational age, severe COVID-19, late-onset sepsis, and hyperbilirubinemia. On the same day, he was administered 1 mL/min oxygen via nasal cannula, intravenous fluid (KAEN-4B), intravenous ceftazidime, intravenous amikacin, and phototherapy. He underwent blood culture examination before being given antibiotics, according to the antimicrobial stewardship protocol. On day 7, his condition worsened; his temperature increased, dyspnea was more severe, and feeding intolerance was observed (Table 2). The blood test results were as follows: activated partial thromboplastin time, 26.3 s (range, 22.9–32.1 s; control, 26.1 s), prothrombin time (international normalized ratio), 1.09 s (0.7–1.2); fibrinogen, 363.6 mg/dL (range, 168–390), and D-dimer, 3,340 ng/mL. Toxoplasmosis, rubella, cytomegalovirus, and herpes simplex virus test results were negative. He was diagnosed as a term infant with an appropriate gestational age, and suspected congenital COVID-19 infection without symptoms, after the results of the COVID-19 RT-PCR test were acquired 3 days later. On day 8, the patient looked more jaundiced, and blood chemistry test results were as follows: total bilirubin, 16.00 mg/dL; direct bilirubin, 2.10 mg/dL; and indirect bilirubin, 13.90 mg/dL.

**Figure 3.**
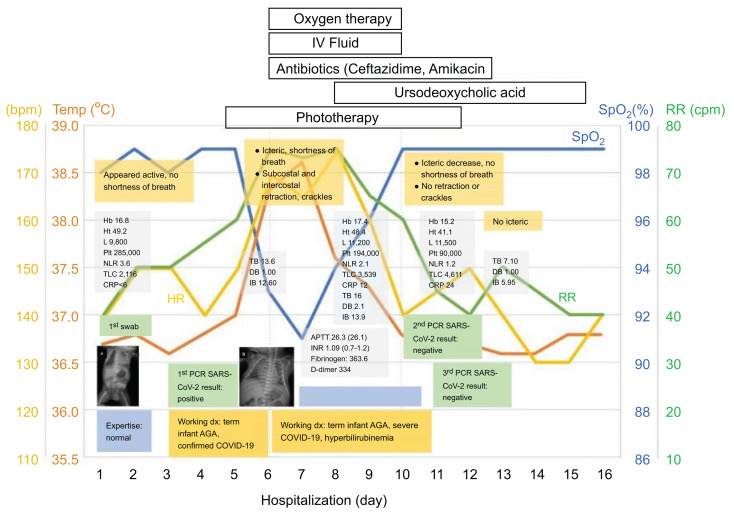
Course of disease. AGA = appropriate for gestational age; APTT = activated partial thromboplastin time; bpm = beats per minute; cpm = cycles per minute; CRP = C-reactive protein; DB = direct bilirubin; Hb = hemoglobin; HR = heart rate; Ht = hematocrit; IB = indirect bilirubin; INR = international normalized ratio; IV = intravenous; L = leukocyte count; NLR = neutrophil lymphocyte ratio; PCR = polymerase chain reaction; Plt = platelet count; RR = respiratory rate; SARS-CoV-2 = severe acute respiratory syndrome coronavirus 2; SpO_2_ = oxygen saturation; TB = total bilirubin; Temp = temperature; TLC, total lymphocyte count. This figure appears in color at www.ajtmh.org.

The patient was administered ursodeoxycholic acid 16 mg twice daily. After 10 days of hospitalization, his condition improved; there was no more dyspnea and his temperature subsided. On days 10 and 11, the RT-PCR SARS-CoV-2 test results were negative. On day 12, the blood culture result was negative, and administration of antibiotics was discontinued. After day 12, the patient’s jaundice was markedly reduced. He was discharged after 16 days of hospitalization, with a final diagnosis of severe COVID-19 and hyperbilirubinemia (Figure 3).

## DISCUSSION

This infant’s status was categorized as congenital COVID-19 based on positive serological confirmation (positive IgG anti-SARS-COV-2 of the mother before delivery and positive RT-PCR SARS-CoV-2 result of the infant). According to the WHO’s latest report in 2021, there was three points of feasibility of vertical transmission. In utero transmission from transplacental transmission can occur from damage to the placenta by an inflammatory reaction, in which cell membrane-associated angiotensin-converting enzyme 2 (ACE-2) and transmembrane protease serine 2 required for SARS-COV-2 have been identified in placental cells. ^12^^,^ ^13^ Transmission during delivery through transvaginal and amniotic fluid cannot be ruled out because these tests are not available. Based on previous reports, transvaginal ^12^ and amniotic transmission occurred only in mothers with severe COVID-19 disease or with high blood viral loads. ^14^ Last, postnatal transmission accounts for the majority of infections reported.

From days 1 to 5, the infant was stable and in good condition, and chest X-ray results were normal. On day 6, he presented with fever, dyspnea, jaundice, lymphopenia, and a neutrophile-to-lymphocyte ratio of > 3.31. Previous studies reported that neonates who were positive for SARS-CoV-2 had symptoms early in life (early onset), and the most common symptoms were fever, dyspnea, and feeding intolerance. The radiographic findings vary. ^6^^,^ ^15^^,^ ^16^ In contrast to previous reports, our patient experienced late-onset, severe COVID-19. The late onset of severe COVID-19 in our patient can be attributed to the natural course of COVID-19 itself, ^17^ and the possible presence of maternal anti-SARS-CoV2 IgG in infants early in life, according to previous research reports. ^12^^,^ ^13^ Whether administering breast milk containing IgM and IgA SARS-CoV-2 antibodies can prevent severe COVID-19 has not been reported and needs more investigation. ^18^

It is sometimes difficult to distinguish neonatal sepsis from COVID-19 because they have similar clinical manifestations. Table 3 shows the clinical manifestations of neonatal sepsis and COVID-19. ^19^ In this case, the patient had clinical manifestations suggestive of sepsis. It is also difficult to rule out bacteria pneumonia at the start of treatment. Therefore, in this case the administration of antibiotic as empirical therapy was still given because we could not rule out bacterial infection. ^20^

**Table 3:** Comparison of neonatal sepsis and COVID-19

Patient no.	Clinical features	Neonatal sepsis	COVID-19
1	Temperature irregularity	Hypothermia (early onset), hyperthermia (late onset)	Hyperthermia/persistent high temperature
2	Change in behavior	Lethargy, irritability, change in tone	Lethargy, irritability, change in tone
3	Skin	Poor peripheral perfusion, cyanosis, mottling, pallor, petechiae, rashes, sclerema, or jaundice singularly or in combination	Rash
4	Feeding problems	Feeding intolerance, vomiting, diarrhea, or abdominal distention	Poor feeding, vomiting, diarrhea
5	Cardiopulmonary	Tachypnea, respiratory distress, tachycardia, hypotension	Cough, rhinitis, respiratory distress
6	Metabolic	Hypoglycemia, hyperglycemia, metabolic acidosis	Metabolic acidosis
7	Focal infections	Cellulitis, impetigo, soft tissue abscesses, omphalitis, conjunctivitis, otitis media, meningitis, or osteomyelitis	No focal infection
8	Laboratory findings	Leukocytosis/leukopenia, neutropenia, ratio of immature to total polymorphonuclear cells > 0.2, increased C-reactive protein, increased procalcitonin	Lymphopenia, thrombocytosis, neutropenia, increased liver function, elevated C-reactive protein, increased D-dimer level, coagulopathy
9	Radiological findings	Normal/infiltrate	Ground-glass appearance

It is common knowledge that the SARS-CoV-2 virus receptor is present in various organs, so the appearance of clinical manifestations can widely vary, although respiratory symptoms are still the main symptom. ^21^

Jaundice in our patient began to appear on day 4 of treatment, and hyperbilirubinemia increased significantly with worsening of the disease. To our knowledge, the presence of neonatal hyperbilirubinemia in neonatal COVID-19 has not been reported previously. Jaundice may occur in severe COVID-19, but is not a typical symptom in newborns. During the first week of life, more than 60% of newborns in developing countries have jaundice. ^22^ The most prevalent risk factors for neonatal jaundice are probably prematurity, hemolytic disease, perinatal infection, and exclusive breastfeeding. ^23^ In this case, possible jaundice may be caused by the COVID-19 infection process, pneumonia, and inadequate early food intake resulting from stress and worries from mothers who have COVID-19. The etiopathogenesis of hyperbilirubinemia resulting from COVID-19 infection is hypothesized to occur by two mechanisms. First, there is impaired active transport of bilirubin uptake into the hepatocytes, or liver injury resulting from expression of the ACE-2 receptor as a target for SARS-CoV-2. Second, there is impaired bilirubin excretion in the presence of cholangiocyte injury resulting from greater ACE-2 expression. ^24^ Meanwhile, from pneumonia itself, jaundice can be caused by two factors—namely, disturbances in the nutritional status of the liver and the presence of toxemia, which causes liver metabolism disorders. ^25^ In our patient, insufficient milk production during the first three days may have played a role in the cause of jaundice in this baby (what we call breastfeeding jaundice). ^26^ Breastfeeding jaundice occurs on days 2 through 5. ^27^^,^ ^28^ A study in a tertiary referral hospital in West Java before the COVID-19 pandemic found that the most common etiology of neonatal jaundice was physiological (23.2%). Other causes included neonatal hepatitis (14.7%), other hemolytic causes (13.7%), infection (12.6%), ABO incompatibility (11.6%), and breastfeeding (11.6%). ^29^

Early adequate feeding is an important and fundamental issue that must be considered in newborns, including when we face new diseases such as COVID-19. In addition to being able to prevent jaundice, early adequate feeding can also prevent clinical worsening of the disease and its associated risk of mortality. ^30^ Therefore, if physicians focus only on new hypotheses about the cause of jaundice, this could result in a harmful situation.

In septic patients, the etiopathology of hyperbilirubinemia usually also results in hemolysis. ^31^ Although the etiology has some differences, both show the same trend of increased bilirubin levels, starting with an increase in indirect bilirubin (unconjugated bilirubin) followed by an increase in direct bilirubin (conjugated bilirubin). This increase is correlated with worsening of the disease. ^24^^,^ ^32^ Our patient was treated with phototherapy and ursodeoxycholic acid, which is the standard drug for cholestasis management. ^33^

There is no specific drug treatment of SARS‐CoV-2 for neonates. ^1^ Symptomatic and supportive treatment, including oxygen supplementation and maintenance of hydration, electrolytes, and acid–base balance, are the mainstay therapies for patients with SARS‐CoV‐2 infection. Supplementation with water and electrolytes should be appropriate to avoid aggravating pulmonary edema and reducing oxygenation. ^7^ Antibiotic therapy for susceptible organism is usually given for 7 to 10 days, and should be discontinued immediately after clinical improvement and negative blood culture results. Neonates with persistent, complicated infections may require a longer duration of antibiotic treatment. It is important to stop antibiotics after a bacterial infection is sufficiently ruled out. In our patient, antibiotics were stopped after 7 days. ^3^^,^ ^9^

The U.S. Food and Drug Administration has approved remdesivir for neonates weighing more than 3,500 g. ^34^ In this case, our patient weighed less than 3,500 g and evidenced hyperbilirubinemia. The administration of remdesivir is not included in the protocol of COVID-19 in children and neonates in Indonesia. Our patient was discharged after resolution of respiratory symptoms and fever, with clinical stability, and after two negative nasal swabs for SARS-CoV-2 acquired 48 hours apart.

We suggest that the screening of infants born from mothers with confirmed COVID-19 or suspected COVID-19 is critical. Neonates who are positive for COVID-19 need thorough observation in the hospital or at home for at least 5 to 7 days to ensure they do not worsen. The judicious use of antibiotics and adequate early feeding are fundamental issues that need attention in newborns with COVID-19 to prevent complications that aggravate the disease.

**Table 1:** Signs, symptoms, and treatment of the patient:

Signs and symptoms, and patient treatment	Hospitalization (day)															
	1	2	3	4	5	6	7	8	9	10	11	12	13	14	15	16
Sign and symptoms																
Temperature, °C	36.7	36.8	36.6	36.8	37.0	38.3	38.6	37.6	37.3	36.8	36.6	36.7	36.6	36.6	36.8	36.8
Dyspnea	–	–	–	–	–	+	+	+	+	–	–	–	–	–	–	–
SpO_2_, %	98	99	98	99	99	93	91	94	96	99	99	99	99	99	99	99
Respiratory rate, cycles/min	40	50	50	55	60	75	73	75	65	60	45	40	50	45	40	40
Heart rate, beats/min	140	150	150	140	150	170	165	175	160	140	145	150	140	130	130	140
Fever	–	–	–	–	–	+	+	+	+	–	–	–	–	–	–	–
Shortness of breath	–	–	–	–	–	+	+	+	+	–	–	–	–	–	–	–
Retraction	–	–	–	–	–	+	+	+	+	–	–	–	–	–	–	–
Crackles	–	–	–	–	–	+	+	+	+	–	–	–	–	–	–	–
Icteric	–	–	–	–	+	+	+	+	+	+	+	+	+	+	+	–
Treatment																
Oxygen therapy	–	–	–	–	–	+	+	+	+	–	–	–	–	–	–	–
Intravenous fluid	–	–	–	–	–	+	+	+	+	–	–	–	–	–	–	–
Antibiotics (ceftazidime, amikacin)	–	–	–	–	–	+	+	+	+	+	+	+	–	–	–	–
Ursodeoxycholic acid	–	–	–	–	–	–	–	+	+	+	+	+	+	+	+	+
Phototherapy	–	–	–	–	+	+	+	+	+	+	+	–	–	–	–	–

– = absent; + = present; SpO_2_ = oxygen saturation.

**Table 2:** Laboratory results

Laboratory examination	Hospitalization day					
	1	5	7	8	11	12
Hemoglobin, g/dL	16.8	–	–	17.4	15.2	–
Hematocrit, %	49.2	–	–	48.4	41.1	–
Leukocyte count, leukocytes/mm^3^	9,800	–	–	11,200	11,500	–
Platelet count, platelets/mm^3^	285,000	–	–	194,000	90,000	–
NLR	3.6	–	–	2.1	1.2	–
TLC, µL	2,116	–	–	3,539	4,611.5	–
CRP, mg/L	< 6	–	–	12	24	–
aPTT, s	–	–	26.3 (26.1)	–	–	–
PT, s; (INR)	–	–	1.09 (0.7–1.2)	–	–	–
Fibrinogen, mg/dL	–	–	363.6	–	–	–
D-dimer, ng/mL	–	–	3,340	–	–	–
Total bilirubin, mg/dL	–	13.6	–	16	–	7.1
Direct bilirubin, mg/dL	–	1	–	2.1	–	1
Indirect bilirubin, mg/dL	–	12.6	–	13.9	–	5.95

aPTT = activated partial thromboplastin time; CRP = C-reactive protein; INR = international normalized ratio; NLR = neutrophil lymphocyte ratio; PT = prothrombin time; TLC = total lymphocyte count.
